# Hemorrhoids—Their Treatment by the Injection Method

**Published:** 1912-01

**Authors:** C. H. Wilkinson

**Affiliations:** Galveston, Texas


### TEXAS MEDICAL JOURNAL.

Established July, 1885

F. E. DANIEL, M. D., - - - - Editor, Publisher and Proprietor

Published Monthly.—Subscription, $1.00 a Year.

VOL. XXVII. AUSTIN, JANUARY, 1912. No. 7.

The publisher is not responsible for the views of the contributors.

### Original Articles.

For Texas Medical Journal.

### Hemorrhoids---Their Treatment by the Injection Method.

BY C. H. WILKINSON, M. D., GALVESTON, TEXAS.

Considering the large number of persons affected with hemorrhoids and the amount of suffering and inconvenience occasioned to the unfortunate possessor by their presence, it seems somewhat remarkable that permanent relief is not oftener sought after by the individual affected, and especially is this so when the means are so attainable and so simple. It is probably correct to state that fully 5 per cent of all individuals who have attained the age of 30 years are, or have been, victims of this troublesome complaint, and as one advances beyond this age the liability to hemorrhoids increases.

Unlike the majority of ailments that affect the human family, the disorder under consideration maintains an obscure and retired existence, rarely being heard of away from home, and only coming into public notice when the ailment becomes so distressingly severe as to reveal its identity to others. This inherent modesty, this secretive nature of hemorrhoids is no doubt one of the causes that withholds the owner of the disease from seeking professional advice concerning them oftener than he ought to.

The individual suffers on in silence, now better, now worse, seeking advice over drug store counters in a whisper, or, oftener still, inducted into answering some one or many of the sure-cure fraudulent advertisements found in the daily papers, until at last he is forced out of his retirement by a severe attack of his trouble to consult a physician.

While modesty is probably a cause of procrastination in the cure of piles, and this is especially so in the case with women, it is by no means the only reason that withholds the sufferer from relief.

From observations I have made upon the subject I am satisfied that the chief and almost the only barrier that stands between the afflicted individual and his physical relief is the inherent fear of being cut.

Thus, the dread of the knife, the fear of pain, is keeping, today, probably a million persons in the United States from seeking relief for a condition that is a perpetual source of annoyance, as well as a great inconvenience to them in the pursuit of their daily avocations, and in many instances by the drain upon their systems is a certain undermining of their physical health. Besides these two objections, the native modesty of the individual afflicted and the fear of steel, a frequent cause for deferring medical treatment in a large proportion of cases is the almost universal excuse of "lack of time" required to undergo a course of treatment.

Hemorrhoids, as a rule, are oftener found prevailing amongst those pursuing active occupations, consequently the objection raised as to lack of time wherein to undergo a course of treatment is by no means unreasonable. The three foregoing causes stated above may, therefore, be considered the principal reasons why so many individuals in every community refrain from coming forward in order to be relieved of piles by operative procedure.

It is the object of this article to demonstrate the fact that a lasting cure of hemorrhoids can be affected without either the use of the knife or of any other cutting instrument, and without the loss of time. The method of procedure alluded to is by the injection method. There are today four principal methods by which a radical cure for hemorrhoids can be effected. The first, most scientific and most formidable is that known as the Whitehead method, by which a circular portion of the mucous membrane and submucous tissue of the lower bowel containing the hemorrhoidal veins are dissected up and cut away, thereby removing the dilated vessels. This operation requires the highest surgical skill for its successful accomplishment, and only experts in such special work should ever attempt its performance.

Instances have occurred as a result of this procedure where persons have been irreparably injured by stricture of the bowel and the destruction of the sphincter muscle of the rectum, or of other important rectal tissues, compelling the use of pads to be worn by the victim of such accidents for the balance of his life, and thereby rendering that man's last end worse than his first.

This operation is tedious, requiring profound anesthesia for its performance; is afterwards painful and necessitates a confinement to bed for several weeks. It is, moreover, dangerous to life as well as to local tissue. The modifications of Whitehead's operation may minimize the dangers, alluded to, but they do not entirely remove them.

The ligating method is probably the most popular means of treating hemorrhoids by the ordinary surgeon. Here again profound anesthesia is requisite, and the pain attending the process of cure is often very severe for many days after the operation.

By this method the piles are drawn down, the mucous membrane nicked around their bases and tightly drawn ligatures are thrown around them. These latter slowly cut their way through the base of the tumors, inflicting a punishment which is born in mind by the patient for the balance of his life. After the piles have sloughed away an ulcer invariably remains which requires weeks to heal. The clamp method, while differing somewhat in its technique from the last named operation, is totally inadequate to reach the vast majority of piles that infest the victim, since often large numbers of them are found occupying the lower bowel too small to be handled with the clamp.

Even when applicable to the case so much of the pile base is left uncut that large ulcers are apt to result and the trouble is, moreover, liable to return. In all the foregoing methods of operating for hemorrhoids general anesthesia is required; pain for many days afterwards is invariably experienced, and dangerous consequences may occur, and, moreover, the patient is compelled to keep his bed sometimes for weeks.

*The Injection Method.*—Contrasted with the foregoing, the method of treating hemorrhoids by injecting them is simple and, as a rule, is safe and painless. It necessitates neither the use of the knife nor of an anesthetic; in 90 per cent of cases is devoid of danger to both the parts operated on as well as to the patient, and in the majority of instances requires no laying up of the individual after operation.

The charges brought against this method that it does not permanently cure, and that it may be followed by injurious consequences are not well founded. I have tried the ligature method and would not adopt it again where phenic acid could be obtained.

Out of over fifty cases I have operated on I can not recall a single instance where a successful result was not obtained by the injection method. But few of my cases experienced pain of any consequence or were compelled to remain in bed a day, except in a few instances where patients came for treatment suffering with an inflamed condition of their piles.

In one instance a rectal abscess was noted, which was soon recovered from, while in several cases small ulcerated spots followed the disappearance of the tumors. These ulcers are to be expected and are of no consequence. They signify the complete removal of the pile, however, and render it certain that the tumor destroyed will never return.

These small sores readily heal under the application of a ten-grain solution of nitrate of silver and their presence in the majority of cases is unknown to the patient.

*Method of Procedure.*—Patients when applying for treatment should be clean and neatly dressed, and should have had a free evacuation of the bowels at the time of application. They should lay upon the left side preferably, with knees well flexed, and should the hemorrhoidal tumor be presented, as invariably it is, a compress soaked in a 2 per cent solution of carbolic acid should be applied to it and allowed to remain thereon for two or three minutes.

If the tumor, however, should not be in sight at the time the individual should be required, if possible, to force it down. Failing to do this the operator should introduce a rectal speculum, preferably a Gants, and thereby bring the tumor into view. Once in sight the operator may follow the directions laid down by Wythe, except that only in a small minority of cases it is necessary to employ cocaine for an anesthetic purpose.

Two formulas are advised by Wythe. The first, a 50 per cent solution of carbolic acid is injected into the pile when rapid sloughing of the tumor, with speedy recovery, is desired; or, when the party affected can afford the time to make repeated subsequent visits to the operator's office, a 10 per cent solution of the acid may be used. Under the latter strength the hemorrhoids are not apt to slough, but the injection is sometimes required to be repeated within a week.

The stronger injection is not desirable, if it can be avoided, as the sloughing that follows will result in an ulcer that may require several weeks to recover from. The formula I have employed has been composed of carbolic acid, glycerine and sterile water, equal parts, of which from 2 to 4 minims are injected into the pile according to the size of the tumor. An ordinary hypodermic syringe will usually suffice for the operation, though an exceptionally long needle may be required to reach a tumor located higher up in the bowel. After the injection has been made the tumor should be forced back above the sphincter muscle of the rectum. The pain that follows the injection of the fluid is slight and momentary, the acid used being itself a local anesthetic, and before the patient leaves the surgeon's office every vestige of it has disappeared. Occasionally a burning sensation may set up in the tumor during the first night following the injection. This, however, rarely is sufficient to warrant an anodyne, yet it is well to provide against this annoyance by prescribing a few 2½-grain tablets of Dover powder and directing one to be taken every half hour should such smarting become distressing.

Exceptionally, a hemorrhoidal tumor may become inflamed after its injection, protruding beyond the verge of the bowel, and become very annoying for five or six days afterwards.

This complication has happened in three of my fifty-five cases, requiring stronger opiates for their relief. By using mild injections this is not apt to occur, and cases should never be operated on if practical to defer them when the hemorrhoids are in an inflammatory condition. The results of the injection are either a rapid shriveling up of the pile tumor, without a break in its mucous covering, or else a slight ulceration follows which is scarcely noticeable to the patient, and which disappears quickly under silver applications made every day or so. Rarely does the individual have to remain in bed during any part of the treatment but keeps at his usual avocation, except in the few instances just alluded to where an inflammatory condition sets up after the injection. The subsequent treatment of hemorrhoids is simple. The patient should be advised to rest in the reclining position for the next six hours and abstain from straining and other physical exercises, in order to prevent the descent of his replaced pile and the setting up of unnecessary inflammatory action. Where these precautions are observed a speedy and satisfactory convalescence is apt to follow, but where they are neglected prolapse of an injected pile may follow and strangulation may result. This is a painful and undesirable consequence which should be corrected as soon as possible by returning the pile to its proper position, and frequently applying to the rectum compresses of lint steeped in hot water.

*Ulcerations.*—After the disappearance of a hemorrhoidal tumor, following the injection, there often remains a small ulceration at the base of the original pile formation. This, however, is of little consequence, but requires attention. The patient follows his usual pursuits unmindful of its presence, yet no case should be discharged from the care of the practitioner while any such ulceration remains.

The cure of such condition, however, is simple and is soon accomplished by applying to the raw surface a solution of nitrite of silver, 10 or 15 grains to the ounce.

These applications should be made every two or three days following the appearance of any ulceration, and for the successful treatment of any of them the patient should be required to return to the surgeon's office punctually at the appointed time for the application, and for the injection of any other pile that may be ready for treatment. During this subsequent treatment the bowels should be kept open by occasional saline cathartics, and in order to facilitate the healing process the individual should be directed to daily flush his lower bowel with an enema consisting of carbolic acid, a dram, and cold water, a pint. Recurrences are not apt to take place after the injection method, since the pile cavity from base to summit is completely obliterated, through the agency of the acid employed in its treatment, but it does not follow that in course of time another hemorrhoidal tumor may not show itself. This happened in two of my cases, but the subsequent pile tumor was insignificant and was located in a different portion of the intestinal tract from the former. When we consider the number of hemorrhoidal veins located in the rectal tissues, each one of which may become liable to pile formation, it is not surprising that other hemorrhoids may appear after the obliteration of the first.

While such recurrences are rare and usually insignificant, it is always better to inform the patient of such possibility, lest in the event of any future pile returning your efforts to relieve him may be regarded as a failure. This recurrence may take place, however, after any other save the Whitehead operation.

It is not claimed by the writer that the injection method is applicable to all cases of hemorrhoids, but in point of simplicity and results in the comparative absence of pain, and the freedom of exercise allowed the patient immediately after the operation it is certainly superior to any other method practiced today for the relief of piles. Andrews (Rectal Surgery) has compiled a list of 3300 cases wherein this method was pursued for anything simulating hemorrhoids, and by every class of operator, skilled and unskilled, with the following results: Deaths, 13; embolism of liver, 8; prostration, 1; abscess of liver, 1; severe hemorrhage, 10; stricture of rectum, 2; carbolic poisoning, 1; failure to cure, 19; sloughing, etc., 35.

Commenting on these results, Tuttle remarks: "Any other surgical operation for hemorrhoids in such inexperienced and unscientific hands would have produced a larger mortality and a longer list of accidents. Can any practitioner cite 3300 cases of hemorrhoids operated on by any other method with only two strictures? The method is well worthy of thorough consideration."

